# Current usage and future trends in gross digital photography in Canada

**DOI:** 10.1186/1472-6920-14-11

**Published:** 2014-01-14

**Authors:** Christopher L Horn, Lawrence DeKoning, Paul Klonowski, Christopher Naugler

**Affiliations:** 1 Department of Pathology and Laboratory Medicine, University of Calgary, 2500 University Dr NW, Calgary, Alberta T2N 1N4, Canada; 2 Calgary Laboratory Services, 9, 3535 Research Rd NW, Calgary, Alberta T2L 2K8, Canada

## Abstract

**Background:**

The purpose of this study was to assess the current usage, utilization and future direction of digital photography of gross surgical specimens in pathology laboratories across Canada.

**Methods:**

An online survey consisting of 23 multiple choice and free-text questions regarding gross digital photography was sent out to via email to laboratory staff across Canada involved in gross dissection of surgical specimens.

**Results:**

Sixty surveys were returned with representation from most of the provinces. Results showed that gross digital photography is utilized at most institutions (90.0%) and the primary users of the technology are Pathologists (88.0%), Pathologists’ Assistants (54.0%) and Pathology residents (50.0%). Most respondents felt that there is a definite need for routine digital imaging of gross surgical specimens in their practice (80.0%). The top two applications for gross digital photography are for documentation of interesting/ complex cases (98.0%) and for teaching purposes (84.0%). The main limitations identified by the survey group are storage space (42.5%) and security issues (40.0%). Respondents indicated that future applications of gross digital photography mostly include teaching (96.6%), presentation at tumour boards/ clinical rounds (89.8%), medico-legal documentation (72.9%) and usage for consultation purposes (69.5%).

**Conclusions:**

The results of this survey indicate that pathology staff across Canada currently utilizes gross digital images for regular documentation and educational reasons. They also show that the technology will be needed for future applications in teaching, consultation and medico-legal purposes.

## Background

Among the many functions of an anatomic pathologist are diagnosis, consultation, documentation, and education. Implicit in these activites is the necessity to document morphological findings both at the macroscopic and microscopic levels [1]. Traditionally, this has been acheived through the process of descriptive prose with inherent idiosyncratic variations in the style, vocabulary and abilities of individual pathologists and other staff performing gross dissection [2]. Often the difference between making a specific diagnosis and a generic pathological process is determined by the gross description including what the gross lesion looked like, where the lesion occurred and how the lesions were distributed [3]. Digital photographs document the true appearances of the pathological changes and may eliminate inaccuracies resulting from variations in descriptive ability [2].

Since pathology is a visual science, the inclusion of quality digital images into lectures, teaching handouts and electronic documents is crucial [4]. When incorporated with synoptic texts, reports are more accurate and concise [5]. In addition, block keys and hand-drawn diagrams can be substituted with digital photographs [2]. In this way, digital photography has changed the face of the pathology report significantly [6], allowing the incorporation of coloured prints of the gross specimen as well as of relevant microscopic features. Some have even gone so far as to suggest that high-resolution digital images could eventually replace word descriptions of macroscopic specimens [7].

In addition to improving current practice, the transition to the digital medium has opened up numerous applications for gross digital pathology such as telepathology, three-dimension image technology and perhaps future automated machine vision systems. Telepathology is already practised to varying degrees worldwide and is primarily used for diagnostic and consultation purposes [8-10]. At a macroscopic level, three-dimensional digital simulations of organs provide an alternative to plastination or formalin-filled pathology pots and may become a possible future necessity for digital diagnostic pathology practice [11]. Additionally, there are several digital imaging applications which are emerging in pathology, such as image analysis using algorithms, coupled with computer-assisted diagnosis and 3D imaging, which have enhanced the field of biomedical informatics [12].

However, in Canada the exact usage and applications of the technology are not very well known. Therefore, standardized methods and applications of these methods are not identified or are thought to be under utilized in Pathology laboratories across the country. A standardized method of obtaining, storing and sharing digital images is needed and can lead to better diagnostic techniques and consultation methods for Pathology diagnosis [6,13-15], however these procedures have yet to materialize [6,16-18]. Additionally, utilizing digital images for teaching and consultation can be more effective for storage purposes and have easier accessibility as compared to traditional print photographs and slides. There is very little data assessing the utilization of gross digital images in Canada. This survey will attempt to give a better overall picture of the current usage and utilization of gross digital images in Canada and examine the perceived future direction of the technology. It may also identify opportunities for further education, research, and software development in this field.

## Methods

To obtain current information regarding the usage of digital imaging from across Canada, a survey was used. A 23 question survey was designed and focused on the type of respondent, knowledge of gross digital photography, current usage, strengths and weaknesses and perceived future direction of digital photography in the pathology laboratory (see Additional file 1). The survey was designed through Survey Monkey and the link to this survey was emailed to all available contacts.

The survey was first distributed to current employees known to be involved in digital pathology practices throughout various sites within the Calgary Laboratory Services (CLS) network. Additionally, surveys were then distributed amongst contacts known to the two main authors of the paper, CH and CN, throughout pathology labs across Canada, with a focus on getting feedback from all parts of the country. These surveys were directed at, but not limited to, pathologists, pathology residents, pathologists’ assistants and laboratory technologists who are known to have direct involvement in handling surgical pathology specimens for preparation and gross dissection. Also, these contacts were asked to distribute the survey link to their colleagues who they could identify as directly involved in handling the surgical pathology specimens in the pathology lab. These individuals were targeted for the survey because they would decide if the specimens required special procedures for the specimens before formalin fixation, including selection for photography. In all, it is estimated that over 100 people were sent the link to the survey by CH, CN and their contacts, which is estimated be approximately 10% of practicing pathology professionals in Canada. This project was approved by the University of Calgary Conjoint Research Ethics Board (Ethics ID#E-25044).

## Results

**Figure 1:**
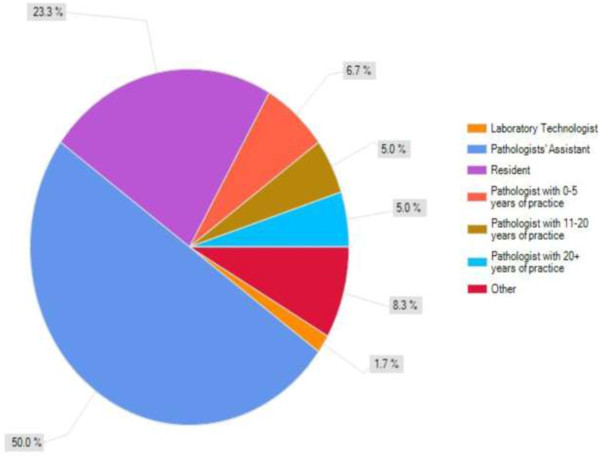
Summary of survey respondents by level of practice.

Overall, we gathered 60 completed survey results (est. 60% response rate) representing most provinces across Canada. The majority of the respondents were pathologists’ assistants (50.0%), pathologists (25.0%) and pathology residents (23.3%), with one medical laboratory technologist, all with various degrees of experience (Figure 1). Of note, no pathologists with six to ten years of experience responded to this survey. We were able to gather responses from most of the areas of the country geographically as follows: British Columbia (2), Alberta (24), Manitoba (1), Ontario (24), Quebec (1), Nova Scotia (4), New Brunswick (1) and Prince Edward Island (1). In all, the respondents represented 36 different institutions, showing also that there were multiple respondents from some of the individual institutions. Two of the respondents did not give us any location where they practice. Some of the respondents practice in community (30.4%) and urban, non-academic settings while most were practicing from Academic centres (58.9%), which are usually indicative of larger, urban centres. A large majority (91.5%) defined gross digital photography as photography of gross specimens with digital camera. Another sizeable number of respondents use digital pathology in their practice (86.2%), with even more stating that gross digital pathology is used at their institution (90.0%). Additionally, they were less likely to have heard or read about gross photography in the literature (60.0%). The large majority of users are the Pathologists (88.0%), and to a lesser extent, pathology residents (50.0%) and pathologists’ assistants (54.0%). However, there seems to be an awareness regarding the technology as a majority of the respondents feel that there is a need for routine digital imaging of gross surgical specimens in their practice (80.0%).

**Figure 2:**
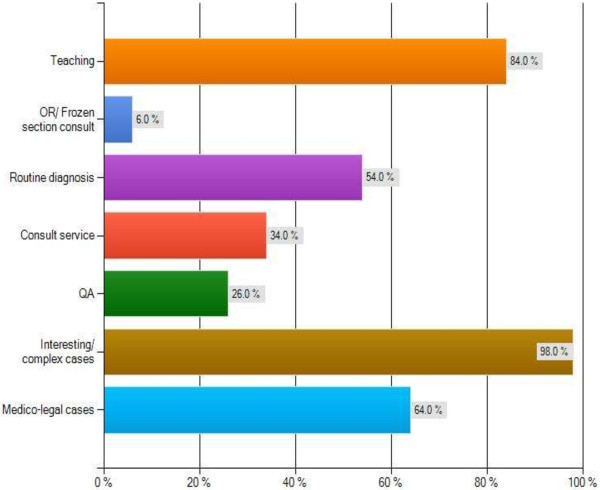
Current applications of gross digital pathology.

**Figure 3:**
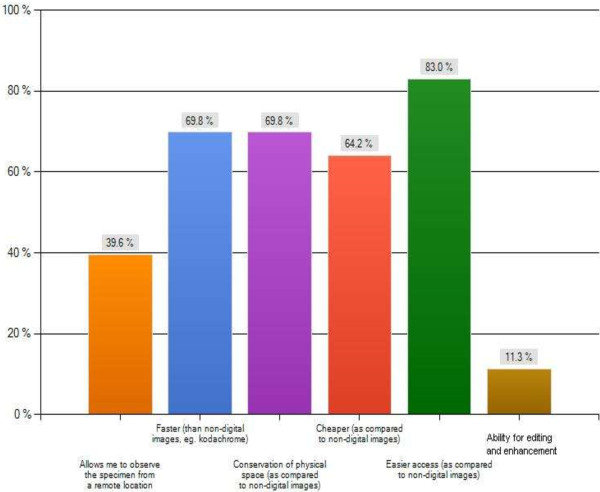
Reported advantages of gross digital pathology.

**Figure 4:**
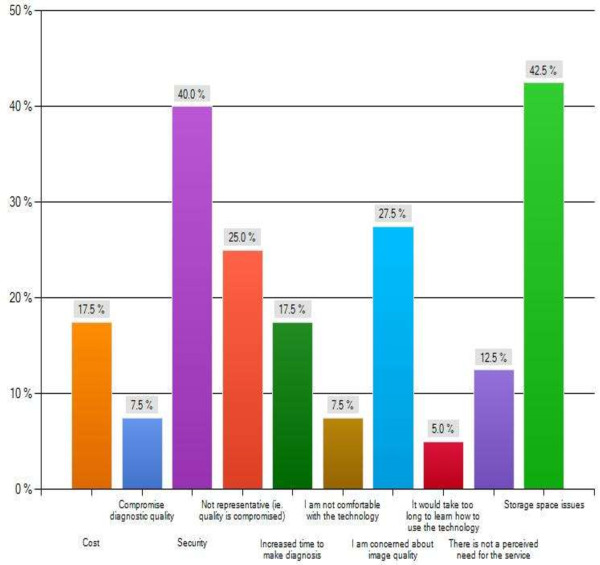
Reported disadvantages of gross digital pathology.

**Figure 5:**
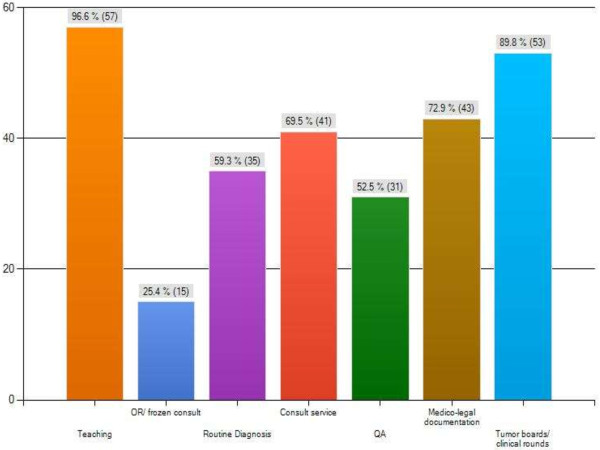
Respondent’s opinion on the future of gross digital pathology.

The major themes of this survey are summarized in Figures 2, 3, 4 and 5 as follows: Current usage (Figure 2), reported advantages (Figure 3) and disadvantages (Figure 4) of gross digital photographs and the applications and future direction of gross digital photography (Figure 5).

Only 30% of respondents report taking gross digital images on a routine basis. Other respondents (31.7%) indicate that gross digital images are only captured for specific types of cases (e.g. gynaecological oncology). Additionally, it is extremely rare (1.7% of respondents) in pathology labs across Canada that ***all*** specimens are selected for gross digital photography.

There is a wide range of the types of cases being selected for gross digital photography. Most respondents stated a need for photography of interesting/complex cases (88.1%), medico-legal documentation (61.0%), complicated cases (55.9%) and cases of a particular type or belonging to a particular subspecialty (e.g. Gynaecological Oncology) (52.5%). Additionally, surgical specimens are photographed at any point of the process, from intact specimens before fixation (79.7%), after fixation (83.1%), after sectioning (81.4%) and of specific features within sections (71.2%).

The gross digital images are usually stored on a central database (79.6%), with other forms of storage also used including individual hard drives (14.8%), and memory sticks (18.5%). Access to these images is varied amongst institutions as pathologists’ assistants (64.9%) and medical staff (57.9%) makes up the majority, while all staff members (26.3%), individual access (8.8%) and administrative staff (3.3%) are the minority. It is also worth mentioning that one respondent did not know who was accessing the images.

Respondents were also asked to expand on the teaching aspect, and in particular - in what teaching environments is it used? The large majority answered clinical rounds/tumour board presentations (90.2%) and resident education (72.5%) as the primary environment. Conference presentation of gross digital images (56.9%) and Continuing Medical Education (CME) type of activities (25.5%) were used, but to a lesser extent. However, a huge majority felt that an on-line digital library of gross digital images would be helpful to review features of challenging or rare cases of gross specimens (96.6%).

An overwhelming amount of the respondents felt that the current application of gross digital photography is for teaching purposes (96.6%) and clinical rounds (89.8%). Additionally, medico-legal documentation (72.9%) and consult services (69.5%) also ranked high. A majority of the respondents also felt that the images were needed for routine diagnosis (59.3%) and Quality Assurance (QA) purposes (52.5%). For example, in breast lumpectomy cases, slices are numbered and photographed. Sections taken are hand-drawn onto the printed photograph for mapping purposes. Also, current utilization of gross digital images for gross-microscopic correlation is used frequently (51.7%) while consultation/second opinion diagnosis usage is rare (18.6%).

## Discussion

Overall, pathology informatics, and specifically gross digital photography has become commonplace in pathology labs across Canada. The majority of the users, which are pathologists, residents and pathologists’ assistants, photograph (document) gross specimens with a digital camera. The images are then commonly stored digitally into a centralized database that is easily accessable for lab personnel. In most institutions, less than 10% of all surgical cases are digitally photographed which are usually classified (documented) as interesting, medico-legal or complicated. After being archived, the digital images are usually accessed by pathologists’ assistants, residents and medical staff for teaching in clinical rounds, resident education and conferences. Additionally, they are accessed for medico-legal and consultation purposes.

There is a wide range of perceived disadvantages to gross digital photography. Storage issues are a major concern among the respondents. Archiving of gross digital images is extremely important for multiple reasons. First, the complexity of archiving will increase with multiple users and acquistion devices plus the ease of retreival and utlization of these images needs to be managed succesfully [12]. Secondly, image files may be quite large, although not to same extent as digital pathology slides. If necessary, this can be circumvented by file compression, the compaction of an image by removal of redundant information, which helps with image processing, storage and transmission [12]. Ideally; acquired pictures should be embedded into pathology reports [19] and then captured directly into a Laboratory Information System (LIS) [12].

Image quality also appears to be of major concern for the respondents. It is essential for gross digital Images to be a good quality representation of the original source [12]. Respondents indicated that there is concern over general image quality and whether this digitial image quality is sufficient for accurate diagnosis (Figure 4). Generally speaking, a good quality image can be taken with a lower end camera producing 3–5 megapixel image [20]. This is necessary to avoid the possible compromise of diagnostic accuracy due to reduced image quality or under representation of the lesion in the digital image [6]. Therefore it is essential to prepare for good gross digital photography by having a well-lit specimen and a clean background [12]. But, most importantly, a high resolution mega-pixel camera with good macro lens with a long barrel for close focusing is essential for optimal gross digital images [12].

Another area of concern appears to be the cost association of gross digital photography. In fact, the most important issues in pathology informatics are challenges associated with the cost of electronic storage [8]. The majority of the costs are associated with building of the new system and training of the pathology health staff which require significant spending [8]. However, after the initial capital investment, the additional operating costs are minimal and can be balanced by the elimination of expenses associated with storage and retrieval of physical (i.e. Kodachrome) images [8]. Reduced costs and extensive applications make the adoption of digital imaging in anatomical pathology laboratories an essential consideration [2,21].

A major concern identified in the survey was an absence of need for gross digital photography. In fact, other studies show that a significant percentage of pathology staff is not in favour [6,8] or not aware of the need of the transition into digital practice. Indeed, in our survey, 10% of the respondents do not use digital photography, indicating reliance on older kodachrome images or no photography used at all at their institution. This may be attributed to a lack of understanding of the applications of gross digital photography and the associated limitations and reduced control over this technology [8]. However, a failure to adopt a digital imaging technology may be considered a deficiency of practice [20]. Indeed, if the reporting pathologist does not employ such a system for proper documentation, back-up or quality assurance it may be considered as negligence in a court of law [2]. However, the recent establishment of pathology informatics interest group that is directed through the Canadian Association of Pathologists serves to implement standards in order to alleviate some of these concerns [22]. The organization was established as an initiative to organize the pathology informatics activities across the entire country [23].

One particular area not specifically addressed by this survey is in relation to ethical issues associated with digital photography. In particular, the issue of fraudulent digital image manipulation has emerged as a particular area of concern [6,12,24,25]. Critics of digital imaging claim that any image manipulation, willingly or unknowingly, alters digital data which causes a misrepresentation of the original information [26]. The critics go on to say that this manipulation is equivalent to scientific fraud [25]. However, fraudulent activities existed long before the arrival of digital imaging and there are undoubtedly easier ways of falsifying scientific data [2].

There are some limitations identified in this survey that warrant discussion. In particular, the sample size of the survey was of concern. We initially tried to reach out to national pathology organizations to distribute this survey to as many pathology laboratory staff as possible, but were rejected for various reasons. Thus, our reliance on the known contacts of the authors and their contacts was the best known option. Despite this limitation, our survey response rate of 60% is well above what is expected for this type of study. Also, as the survey was distributed to current employees known to be directly involved in gross digital photography, this could be considered a bias, as the respondents are more familiar with this topic and may not be representative of the pathology community at large. Additionally, as with other voluntary studies, not all individuals are inclined to participate. In general, only those with strong opinions are more likely to respond. In this case, probably those respondents who have either strongly positive or negative experiences with gross digital photography were most likely to respond to the survey.

## Conclusions

There is no doubt as to the current usefulness and future applications of gross digital photography in the pathology lab. This survey has shown that the technology is being utilized consistently by pathologists, residents, pathologists’ assistants for documentation and educational purposes of diagnostic specimens. According to this survey, the majority of pathology lab personnel in Canada feel that gross digital photography should be utilized for educational purposes including teaching and round presentation, which is consistent with previous reports [6]. Also, it is needed for medico-legal documentation, consultation, QA and routine diagnostic services. Respondents of the survey identify future applications of gross digital photography in Canada as teaching, consultation and medico-legal documentation. There is less excitement indicated for telepathology, and three-dimensional digital pathology methods, both of which were previously identified as an emerging trend in pathology informatics [6,8-12]. This survey identifies the current and future trends of gross digital photographs in Canada and can be used to help laboratory personnel in setting up and organizing their databanks. Since the future trends in Canada are now known, databanks can be designed to anticipate future needs.

## Competing interests

The authors declare that they have no competing interests.

## Authors’ contributions

CH and CN conceived of the study. CH collected the data, contributed to the study design, and drafted the manuscript. CN, LD and PK contributed to the study design. All authors contributed to revisions, read and approved the final manuscript.

## Pre-publication history

The pre-publication history for this paper can be accessed here:

http://www.biomedcentral.com/1472-6920/14/11/prepub

## Supplementary Material

Additional file 1 Survey regarding the utilization and application of gross digital images in the pathology laboratory. Click here for file
