# Supplementary material for: Current usage and future trends in gross digital photography in Canada
Source: BMC Med Educ. 2014 Jan 14;14:11. doi: 10.1186/1472-6920-14-11 (PMC3909320; doi:10.1186/1472-6920-14-11)
Supplement: Additional file 1 — Survey regarding the utilization and application of gross digital images in the pathology laboratory. [file 1472-6920-14-11-S1.doc]

**Additional file 1 - Survey Regarding the Utilization and Application of Gross Digital Images in the Pathology Laboratory.**

1. Level of Practice

| Laboratory Technologist  Pathologists' Assistant  Resident  Pathologist with 0-5 years of practice  Pathologist with 6-10 years of practice  Pathologist with 11-20 years of practice  Pathologist with 20+ years of practice |
| --- |
| Other (please specify) |

2. What is the name of your institution?

3. What type of centre do you practice?

| Academic  Urban non-academic  Community |
| --- |
| Other (please specify) |

4. Do you use digital pathology in your practice?

| Yes  No |
| --- |

5. Is digital pathology of gross specimens (Gross Digital Photography) used in at your institution?

| Yes  No |
| --- |

6. How do you define gross digital pathology?

| photography of gross specimens with digital camera  photography of microscopic images with digital camera  storage of pathology images into digital format  sharing of pathology digital images for teaching/ consultation of diagnosis |
| --- |
| Other (please specify) |

7. Are you aware of/ read about gross digital pathology in the literature?

| Yes  No |
| --- |

8. Do you see a need for routine digital imaging of gross surgical specimens in your practice?

| Yes  No |
| --- |

9. If yes to Question 8, who is the primary user of the gross digital images at your institution?

| Laboratory Technologists  Pathologists' Assistants  Residents  Pathologists  Clinical Colleagues  Scientists |
| --- |
| Other (please specify) |

10. If yes to question 8, in what capacity is the technology used? (choose all that apply)

| Teaching  OR/ Frozen section consult  Routine diagnosis  Consult service  QA  Interesting/ complex cases  Medico-legal cases |
| --- |
| Other (please specify) |

11. How often do you use Gross Digital Photography?

| Every case  Routinely  Daily  Rarely  Never  Only for certain types of cases |
| --- |

12. Estimate which percentage of Gross specimens are photographed

| <10%  25%  50%  75%  100% |
| --- |

13. What types of Gross specimens are digitally photographed?

| All cases  All cases of a particular type or for a particular subspecialty  Interesting cases  Complicated cases only  Medico-legal cases |
| --- |

14. What sorts of Gross Digital Images are taken? (choose all that apply)

| Of intact gross specimen (before fixation)  Of intact gross specimen (after fixation)  Of specimen after it has been sectioned  Of specific features within section(s) |
| --- |
| Other (please specify) |

15. If yes to question 8, how are the images stored?

| Central database  Individual hard drive  Memory card/ stick etc. |
| --- |
| Other (please specify) |

16. Who has access to your Digital Gross Images?

| Only I do  All employees  Residents  Medical Staff  Pathologists' Assistants |
| --- |
| Other (please specify) |

17. If yes to question 8, what are your observed advantages in using Gross Digital Images (please select all that apply).

| Allows me to observe the specimen from a remote location  Faster (than non-digital images, eg. kodachrome)  Conservation of physical space (as compared to non-digital images)  Cheaper (as compared to non-digital images)  Easier access (as compared to non-digital images)  N/A |
| --- |
| Other (please specify) |

18. If you use gross digital images for teaching, in what teaching environments is it used?

| CAP PIP or ASCA Check Path or CME activities  Conferences  Resident Education  Clinical Rounds/ Tumor boards |
| --- |
| Other (please specify) |

19. What do you think should be the application(s) of Gross Digital Pathology? (choose all that apply)

| Teaching  OR/ frozen consult  Routine Diagnosis  Consult service  QA  Medico-legal documentation  Tumor boards/ clinical rounds |
| --- |
| Other (please specify) |

20. Have you ever requested a second opinion/ consult using a Gross Digital Image?

| Yes  No  N/A |
| --- |

21. In your opinion, what are the disadvantages in using Gross Digital Images? (select all that apply)

| Cost  Compromise diagnostic quality  Security  Not representative (ie. quality is compromised)  Increased time to make diagnosis  I am not comfortable with the technology  I am concerned about image quality  It would take too long to learn how to use the technology  There is not a perceived need for the service  Storage space issues |
| --- |
| Other (please specify) |

22. Would you favour having an on-line digital library to review features of challenging or rare cases of gross specimens?

| Yes  No |
| --- |

23. Do you perform gross-microscopic correlation with the aid of Gross Digital Images?

| Yes  No  N/A |
| --- |
| Additional Comments |
